# Supplementary material for: Causal Relationship between Aging and Anorexia Nervosa: A White-Matter-Microstructure-Mediated Mendelian Randomization Analysis
Source: Biomedicines. 2024 Aug 16;12(8):1874. doi: 10.3390/biomedicines12081874 (PMC11351342; doi:10.3390/biomedicines12081874)
Supplement: Supplementary file 1 [file biomedicines-12-01874-s001.zip › Supplementary Figure/Figure S8/Leaveoneout plot of MR analysis for TL to ubm-b-1556.pdf]

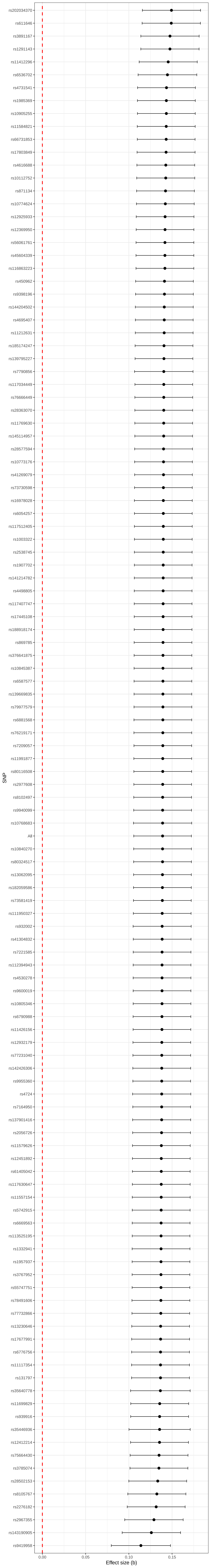
rs202034370
rs611646
rs3891167
rs1291143
rs11412296
rs6536702
rs4731541
rs1985369
rs10905255
rs11584821
rs66731853
rs17803849
rs4616688
rs10112752
rs871134
rs10774624
rs12925933
rs12369950
rs56061761
rs45604339
rs116863223
rs450962
rs9398196
rs144204502
rs4695407
rs11212631
rs185174247
rs139795227
rs7790856
rs117034449
rs76666449
rs28363070
rs11769630
rs145114957
rs28577594
rs10773176
rs41269079
rs73730598
rs16978028
rs6054257
rs117512405
rs1003322
rs2538745
rs1907702
rs141214782
rs4498805
rs117407747
rs17445108
rs188918174
rs869785
rs376641875
rs10845387
rs6587577
rs139669835
rs79977579
rs6881568
rs76219171
rs7209057
rs11991877
rs80116508
rs2977608
rs8102497
rs9940099
rs10768683
All
rs10840270
rs80324517
rs13062095
rs182059586
rs73581419
rs111950327
rs932002
rs41304832
rs7221585
rs112394943
rs4530278
rs9600019
rs10805346
rs6790988
rs11426156
rs12932179
rs77231040
rs142426306
rs9955360
rs4724
rs7164950
rs137901416
rs2056726
rs11579626
rs12451892
rs61405042
rs117630647
rs11557154
rs5742915
rs6669563
rs113525195
rs1332941
rs1957937
rs3767952
rs55747751
rs78491606
rs77732866
rs13230646
rs17677991
rs6776756
rs11117354
rs131797
rs35640778
rs11699829
rs939916
rs35446936
rs12412214
rs75664430
rs3785074
rs28502153
rs8105767
rs2276182
rs2967355
rs143190905
rs9419958
SNP
0.00
0.05
0.10
0.15
Effect size (b)
